# Construction of miRNA‐lncRNA‐mRNA co‐expression network affecting EMT‐mediated cisplatin resistance in ovarian cancer

**DOI:** 10.1111/jcmm.17477

**Published:** 2022-07-10

**Authors:** Amirhosein Naghsh‐Nilchi, Laleh Ebrahimi Ghahnavieh, Fariba Dehghanian

**Affiliations:** ^1^ Department of Cell and Molecular Biology and Microbiology, Faculty of Biological Science and Technology University of Isfahan Isfahan Iran

**Keywords:** cisplatin resistance, co‐expression network, epithelial‐mesenchymal transition, ovarian cancer, prognostic markers

## Abstract

Platinum resistance is one of the major concerns in ovarian cancer treatment. Recent evidence shows the critical role of epithelial–mesenchymal transition (EMT) in this resistance. Epithelial‐like ovarian cancer cells show decreased sensitivity to cisplatin after cisplatin treatment. Our study prospected the association between epithelial phenotype and response to cisplatin in ovarian cancer. Microarray dataset GSE47856 was acquired from the GEO database. After identifying differentially expressed genes (DEGs) between epithelial‐like and mesenchymal‐like cells, the module identification analysis was performed using weighted gene co‐expression network analysis (WGCNA). The gene ontology (GO) and pathway analyses of the most considerable modules were performed. The protein–protein interaction network was also constructed. The hub genes were specified using Cytoscape plugins MCODE and cytoHubba, followed by the survival analysis and data validation. Finally, the co‐expression of miRNA‐lncRNA‐TF with the hub genes was reconstructed. The co‐expression network analysis suggests 20 modules relating to the Epithelial phenotype. The antiquewhite4, brown and darkmagenta modules are the most significant non‐preserved modules in the Epithelial phenotype and contain the most differentially expressed genes. GO, and KEGG pathway enrichment analyses on these modules divulge that these genes were primarily enriched in the focal adhesion, DNA replication pathways and stress response processes. ROC curve and overall survival rate analysis show that the co‐expression pattern of the brown module's hub genes could be a potential prognostic biomarker for ovarian cancer cisplatin resistance.

## 1 INTRODUCTION

Ovarian cancer is one of the deadliest gynaecological malignancies with a low survival rate (Near 15% 5‐year survival for stage IV).^1^ Ovarian cancers are classified as non‐epithelial and epithelial ovarian cancers (EOC). EOC is associated with ovarian cancer‐related deaths.^2^ About 25% of patients with ovarian cancer are resistant to platinum‐based therapy.^3^ Furthermore, about 80% of patients suffer from recurrence of ovarian cancer, and these tumours are typically platinum‐resistant, which leads to chemotherapy failure.^3^, ^4^ So, it is crucial to overcome this resistance in ovarian cancer cells. Cis‐diamminedichloroplatinum (cisplatin) is a platinum‐based chemotherapy drug that treats various cancers like bladder, ovarian, lung, breast and brain cancers.^5^, ^6^ Cisplatin forms DNA adducts involved in activating DNA damage recognition, DNA repair and apoptosis signalling pathways.^7^ Two mechanisms have been suggested for platinum resistance in cancer cells. In the first mechanism, cisplatin uptake decreases while its detoxification increases. The second mechanism is the activation of anti‐apoptotic pathways like NF‐κB and MAPK pathways after treatment.^8^, ^9^, ^10^, ^11^

Recent evidence shows the association of EMT with cellular resistance to cisplatin. During the EMT process, epithelial cells lose the polarized epithelial structure and transform into moving mesenchymal cells, increasing their invasiveness. It has been shown that EMT is associated with drug resistance and apoptosis scape in a variety of cancer types.^12^, ^13^, ^14^, ^15^ Many observations indicate a link between drug resistance and EMT in various cancers like colorectal,^16^ breast^17^, ^18^ and ovarian.^19^, ^20^, ^21^, ^22^, ^23^, ^24^, ^25^ Furthermore, Miow et al.^7^ discovered that epithelial‐like ovarian cancer cell lines exhibit resistance to cisplatin treatment, along with NF‐κB activation and apoptotic impairment. Identification of molecular mechanisms which are involved in this process would be helpful.

Today, co‐expression network analysis is used for underlying the regulatory mechanisms relating to the specific biological processes. The WGCNA is a powerful method for analysing gene expression data, discovering modules of highly related genes and connecting each module to sample traits.^26^, ^27^ This tool constructs a co‐expression network based on the expression profile similarities in samples.^28^ WGCNA hierarchical clustering methods use holistic gene expression information to discover gene network signatures in a phenotype, which helps us to reduce bias.^29^

In this study, we applied WGCNA to the expression profile of cisplatin‐treated ovarian cancer cell lines to identify critical modules in treated cell lines with epithelial status compared to mesenchymal cell lines. These modules were closely associated with cisplatin resistance in ovarian cancer cell lines. The analysis of co‐expression networks may decipher new insights into molecular mechanisms and signalling pathways of drug resistance in ovarian cancer to improve its prognosis and treatment.

## 2 MATERIALS AND METHODS

### 2.1 Acquisition of microarray datasets

**FIGURE 1:**
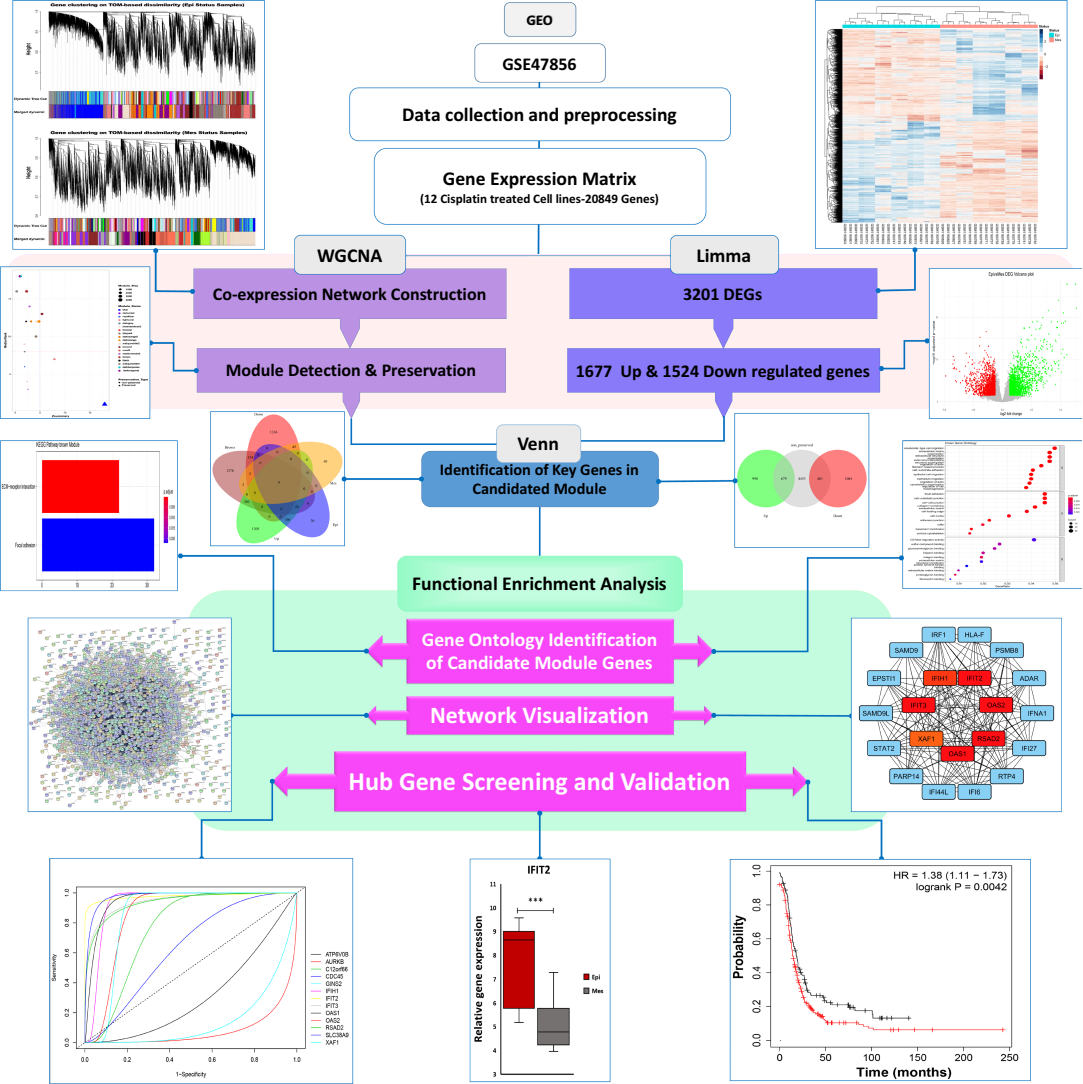
Schematic workflow of overall in silico steps

The flow chart of our study is shown in Figure 1. Raw CEL files of Microarray dataset GSE47856 from the NCBI Gene Expression Omnibus (GEO) database were collected.^7^ The dataset platform was GPL6244 Affymetrix Human Gene 1.0 ST Array [HuGene‐1_0‐st], including 46 different human ovarian carcinoma cell lines divided into two groups based on Cisplatin treatment. The 16 Cisplatin‐treated cell lines with three biological replicates were selected for further analysis.

### 2.2 Microarray data preprocessing and DEGs identification

A total of 48 samples in CEL format were simultaneously pre‐processed using Robust Multi‐chip Analysis (RMA) function for background adjustment, quantile normalization and summarization.^30^, ^31^ To come by the highest possible level of data quality and to eliminate mistargeted and nonspecific probes on the microarrays, the Principal Component Analysis (PCA) was used to identify and remove outlier samples from the dataset.^32^ The multiple probes measure the expression of a given gene, and it is necessary to collapse the multiple probe sets to the same gene by applying the collapseRows function, which was reported as an effective method previously (MaxMean was used for collapsing rows).^33^ Finally, 20,849 genes were used as input in the DEGs and co‐expression network analyses. The limma package was used for DEGs analysis through linear modelling and empirical Bayes methods.^34^ The criteria considered for DEG extraction were as |log2 fold change| ≥ 0.58 and adjusted *p*‐value <0.05.

### 2.3 Construction of a signed‐hybrid weighted gene co‐expression network

To construct the co‐expression networks, we used the WGCNA package. A signed‐hybrid weighted gene co‐expression network was built based on Mesenchymal and Epithelial gene expression. The pickSoftThreshold function of the WGCNA package was used to set soft threshold power β as a tradeoff between scale‐free topology and mean connectivity for Mesenchymal and Epithelial.

### 2.4 Module detection

#### 2.4.1 Generating adjacency and TOM similarity matrices

Based on the selected soft‐power, calculation of the adjacency matrix into a topological overlap matrix (TOM) was accomplished to minimize the effects of noise and spurious associations. Based on the TOM dissimilarity, hierarchical clustering was exerted to classify highly co‐expressed genes as dense interconnected branches of the tree (dendrogram) into the same modules and extracted through the dynamic hybrid tree‐cutting algorithm. Modules with high eigengene correlation were merged using the mergeCloseModules function (cutHeight = 0.3, corresponding to correlation of 0.7).^35^ Application of eigengenes in WGCNA would be in modules summarization and measuring module memberships (kME) to earn suitable target genes. These genes are recognized as connectivity based on eigengenes, which are calculated by the moduleEigengenes function. Eigengenes would be conducted as the first principal component of each module leading to the weighted average of the module's co‐expression profiles by summarizing and comparing them.^36^, ^37^

#### 2.4.2 Module preservation analysis

To assess critical modules in the Epithelial network compared to the Mesenchymal network, the modulePreservation analysis of the WGCNA package was used based on Zsummary and medianRank benchmark. While the value of Zsummary depends on module size with a positive correlation, the medianRank was employed to compare the preservation details of modules in different sizes. In other words, a given module with a lower value of Zsummary and higher medianRank manifests the module which is non‐preserved between the Epithelial and Mesenchymal statuses. Our study determined preserved modules with higher density and connectivity by Zsummary >5 and MedianRank ≤8.^35^

#### 2.4.3 Gene ontology and pathway enrichment analysis of the candidate module

Significant up‐ and down‐regulated genes in the four non‐preserved modules were identified by the VennDiagram package version 1.6.20 in R, to candidate module with the most fluctuated gene signature related to ovarian cancer cells with cisplatin resistance.^38^ Afterwards, significantly implicated GO and KEGG pathways of the candidate module's genes were identified by the clusterProfiler^39^ package in R (adjusted *p*‐value <0.05).

### 2.5 Construction of protein–protein interaction Network

The protein–protein interaction (PPI) network of the non‐preserved module was constructed using the STRING tool in Cytoscape version 3.7.2.^40^ The potential interaction between genes at the protein level predicts protein interactions and the weight of each edge (line) in the PPI network. The criteria used for the network is a combined score greater than 0.4. The molecular complex detection (MCODE) analysis was used to identify the significant clusters of the candidate module PPI network with degree cut‐off 2, max depth 100, k‐core 2 and node score cut‐off 0.2.^41^ MCODE's highest‐ranked cluster was screened by the cytoHubba Cytoscape plugin, using Maximal Clique Centrality (MCC) parameter to detect hub genes.^42^

### 2.6 Reconstruction of miRNA‐lncRNA‐TF‐hub gene co‐expression network

The significantly differentially expressed miRNAs, lncRNAs and transcription factors (TF), which have co‐expression correlation with hub genes, were identified. The co‐expression network was visualized using Cytoscape version 3.7.2.

### 2.7 Hub gene screening and validation

Among the candidate hub genes, the Kaplan–Meier survival curve and the receiver operating characteristic (ROC) curve were used to predict the potential ability of each gene to be independent predictors using the KM‐plotter^43^ and easyROC (version 1.3.1)^44^ as interactive web tools. The platin‐based treatment survival curves for ovarian cancer patients were plotted using data from GEO, EGA and TCGA databases to confirm the genes contributing to survival. Single‐gene survival analysis was executed on hub genes in non‐preserved modules as the significant prognostic module. Finally, univariate and multivariate Cox regression analyses were performed on processed RNA‐seq transcription profiling data (GSE149146) provided by Gallon et al.^45^ from the GEO database to assess whether these hub genes could be independent prognostic biomarkers of ovarian cancer chemotherapeutic resistance.

## 3 RESULTS

### 3.1 Uncovering of modules related to cisplatin resistance in ovarian cell lines

**FIGURE 2:**
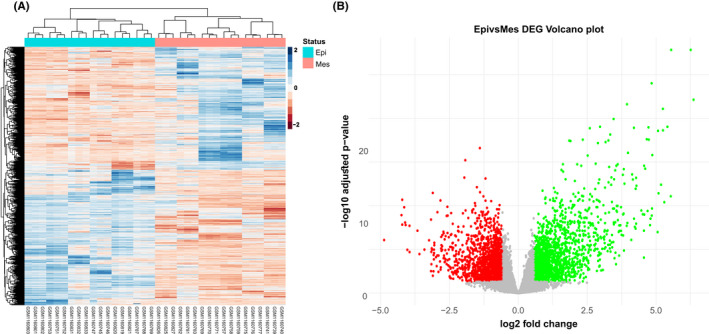
The heatmap and volcano plots indicate 3201 DEGs including 1677 up‐regulated and 1524 down‐regulated genes. (A) Differentially expressed genes (DEGs) clustering heatmap, blue indicates Epithelial, and pink indicates Mesenchymal group. (B) In the DEGs' volcano plot, the *x*‐axis represents log2 fold change, and the *y*‐axis indicates −log10 adjusted *p*‐value. Each dot represents one gene with detectable expression in both Mesenchymal and Epithelial groups. Green and red dots show significantly up and down‐regulated genes based on the given criteria (|log2FC| ≥ 0.58 and adjusted *p*‐value <0.05)

We obtained a normalized expression profile data matrix of the GSE47856 GEO dataset containing 36 Ovarian Cell line samples (12 cell types with triple biological replication treated with cisplatin) and 20,849 expressed genes. Three thousand two hundred and one DEGs were identified based on the screening criteria as |log2FC| ≥ 0.58 and adjusted *p*‐value <0.05, including 1677 up‐regulated and 1524 down‐regulated genes. The volcano plot and heatmap of differentially expressed genes were illustrated in R by ggplot2 package version 3.3.5^46^ (Figure 2).

**FIGURE 3:**
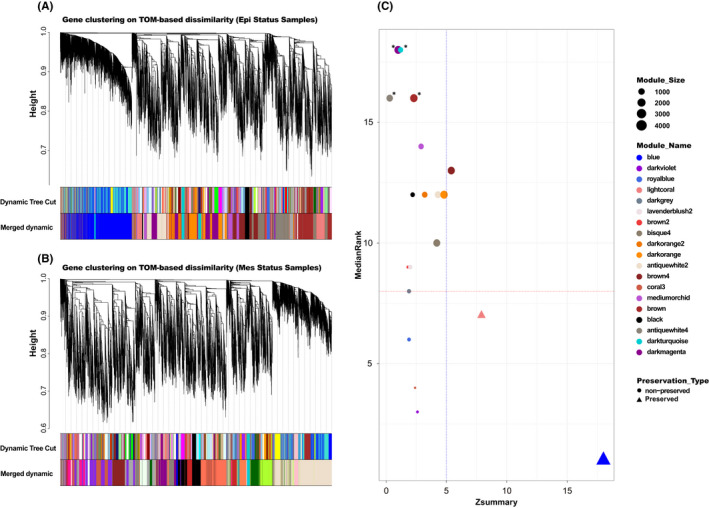
Four non‐preserved modules are identified in Epithelial cell lines through network preservation analysis based on the MedianRank and Zsummary criteria. Hierarchical clustering dendrograms of identified co‐expressed genes in (A) Epithelial and (B) Mesenchymal modules based on a dissimilarity measure (1‐TOM). Each horizontal‐coloured bar represents highly interconnected genes that correspond to each module. (C) The module preservation analysis of epithelial network as reference and Mesenchymal as test data; each point represents a module, labelled by different colours. Four non‐preserved modules were considered (indicated by *). The dashed blue and red lines indicate Zsummary = 5 and MedianRank = 8 thresholds

An optimal soft‐thresholding power is primarily required to construct a weighted co‐expression network in which co‐expression similarity was fetched up adjacency calculation. Thus, we utilized network topology analysis for various soft thresholding powers to have the network's relatively balanced scale‐free and mean connectivity by WGCNA package (Figure S1A,B). We used WGCNA‐recommended power based on the number of samples (power 9). This power was chosen to produce expressed genes’ hierarchical clustering trees based on TOM dissimilarity. Subsequently, the WGCNA gene clustering was performed to divide genes into various modules with similar expression and phenotypes tendency association. Two different hierarchical clustering trees for Mesenchymal and Epithelial cell lines were generated. Afterwards, modules with eigengene correlation above 0.7 were merged (Figure S1C,D), which resulted in 20 modules in both Mesenchymal and Epithelial. Every similarly expressed gene, represented as tightly connected leaves on the dendrogram, displayed a gene module. The resulting gene dendrogram with respective module colours is shown in Figure 3A as Epithelial and Figure 3B as Mesenchymal clustering dendrogram. The number of genes per module has been displayed in Table S1. Mesenchymal and Epithelial status were used as independent variables to calculate the module's tendency to ovarian cisplatin resistance initiation and progression.

### 3.2 Four non‐preserved modules are identified in epithelial cell lines through network preservation analysis

We used the Epithelial data set as the reference and the Mesenchymal data set as the test for network preservation analysis. Due to the high difference between Epithelial and Mesenchymal expression, we expected most modules to be non‐preserved. In this sense, module preservation indicates which module is related explicitly to Epithelial status. Preservation analysis presented four non‐preserved modules, including antiquewhite4, brown, darkmagenta and darkturquoise modules with Zsummary <2.5 and medianRank >15 (Figures 3C and S2; Table S2). The alteration of connectivity patterns in the non‐preserved modules may be related to drug resistance status and recurrence of ovarian cancer. We hypothesized that non‐preserved modules in Epithelial cell lines might highlight dysregulated pathways in the drug resistance compared to the sensitive network. Figure 3C shows the preservation statistics of Epithelial modules in the Mesenchymal network.

### 3.3 Antiquewhite4, brown and darkmagenta modules covering the most number of DEGs among non‐preserved modules

**FIGURE 4:**
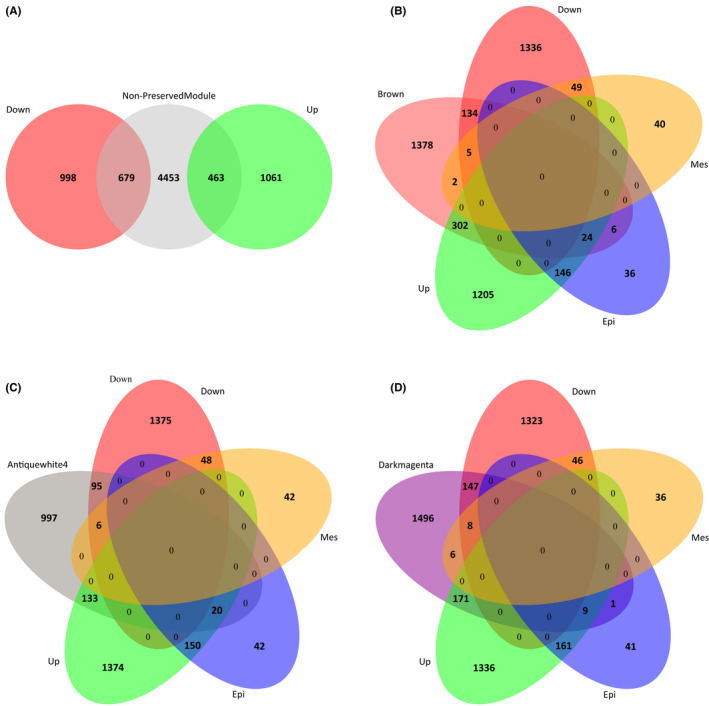
Genes in non‐preserved modules show overlap with the significant up, and down‐regulated gens (DEGs). (A) The significant 463 up and 679 down‐regulated genes were screened by the Venn diagram for non‐preserved modules. Overlapped Up and down‐regulated genes in (B) Brown, (C) Antiquewhite4 and (D) Darkmagenta modules with DEGs and Mesenchymal and Epithelial markers. (Up‐regulated, down‐regulated genes shown in green and red; Mesenchymal, Epithelial markers shown in yellow and blue, respectively)

A total of 679 and 463 genes showed significant up/down‐regulation in non‐preserved modules, respectively (Figure 4A). After discrete assessment of overlapped DEGs among four non‐preserved modules (Figure S3), Epithelial and Mesenchymal markers discovered by Miow et al.^7^, ^47^ were also captured to choose critical modules in cisplatin resistance status. Figure 4B–D represent the 326, 153 and 180 up, 139, 101 and 155 down‐regulated genes, and 30, 20 and 10 Epithelial markers belonging to the brown, antiquewhite4 and darkmagenta non‐preserved modules, respectively.

### 3.4 Enrichment analysis in antiquewhite4, brown and darkmagenta non‐preserved modules implies cancer progression

**FIGURE 5:**
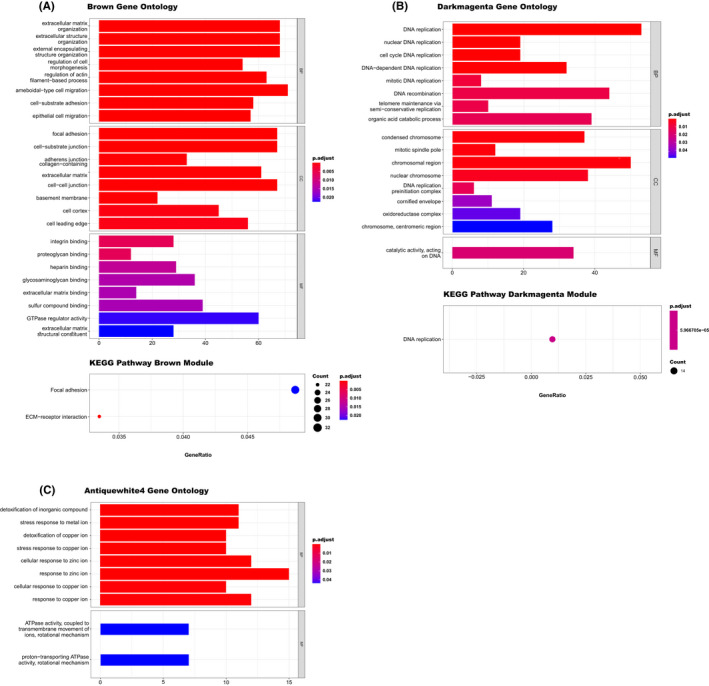
GO functional enrichment analyses of genes in the candidate modules suggest specific roles in epithelial cell migration, extracellular matrix organization, cell–cell junction, DNA replication processes and stress response. GO term and KEGG pathway enrichment analysis results for (A) Brown, (B) Darkmagenta and (C) Antiquewhite4 modules. The bar plot depicts the biological process (BP), molecular function (MF) and respective cellular component (CC) of the GO terms (up). The bubble plot illustrates the most significant and top KEGG pathways (down). The *y*‐axis shows the GO and KEGG pathway terms and the number of genes on the *x*‐axis. The region of bars and bubbles is significantly proportional to the number of genes in a given GO term or KEGG pathway. The adjusted *p*‐value of each term is coloured according to the legend

GO function and KEGG pathway enrichment analyses were performed to assess the function of co‐expressed genes in the antiquewhite4, brown and darkmagenta modules. As shown in Figure 5A, the brown module is enriched in epithelial cell migration, extracellular matrix organization, ameboidal‐type cell migration, focal adhesion, cell−substrate junction, cell–cell junction and binding functions such as integrin, heparin, and glycosaminoglycan binding, and KEGG pathway including focal adhesion and ECM receptor interaction pathways. The GO and KEGG enrichment analysis of the darkmagenta module containing DNA replication processes, catalytic activity acting on DNA and DNA replication pathway are illustrated in Figure 5B. Antiquewhite4 module is primarily enriched in stress response biological processes and proton and ions transporting ATPase activity function shown in Figure 5C.

### 3.5 Candidate modules PPI network construction illustrates key genes in cancer and drug resistance

**FIGURE 6:**
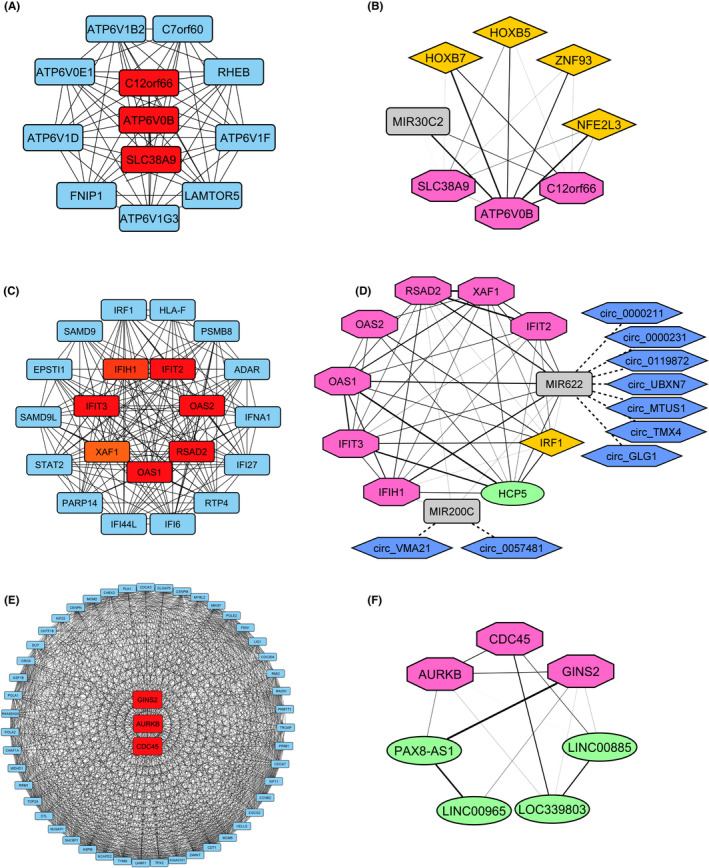
Comprehensive analysis of the candidate modules protein–protein interaction (PPI) network explicit hub genes in cisplatin resistance. The significant modules were identified from the PPI network using the MCODE method. Nodes stand for proteins, and each edge for the interaction between them. The importance of nodes in the network is displayed in two different colours. Red shows top‐ranked proteins by cytoHubba, and blue represents nodes interacting with hub genes. (A) CytoHubba and (B) co‐expression network of miRNA‐TF with hub mRNAs of Antiquewhite4 module. (C) CytoHubba and (D) co‐expression network of miRNA‐lncRNA‐TF with hub genes of Brown module. (E) CytoHubba and (F) co‐expression network of lncRNA with Darkmagenta module's hubs. Grey rectangle, green oval, yellow diamond, blue hexagon and pink octagonal nodes stand for miRNAs, lncRNA, TF, circRNA and mRNAs. The transparency of each edge shows the weight of the co‐expression between each node. Dash line edges show the regulatory interaction between circRNAs and miRNAs

The antiquewhite4, brown and darkmagenta module genes were used to construct the physical and functional associations of the proteins. The antiquewhite4, brown and darkmagenta module PPI networks comprise 1041, 1594 and 1579 nodes as proteins and 3109, 7440 and 9331 edges as their interactions, extracted from the STRING tool in Cytoscape software. The top‐scored interpreted clusters as closely interlinked regions of the PPI network ranked by Cytoscape plugin MCODE were chosen for further analysis (antiquewhite = 11.818, brown = 20.435, darkmagenta = 39.143). The top MCODE cluster with detailed topological parameters is represented in Table S3. Antiquewhite4 top cluster consists of 12 nodes and 65 edges (Figure 6A), brown1 consists of 24 nodes and 235 edges (Figure 6C), and finally, darkmagenta contains 50 nodes and 959 edges (Figure 6E).

Chromosome 12 open reading frame 66 (C12orf66), ATPase H+ Transporting V0 Subunit B (ATP6V0B) and solute carrier family 38, member 9 (SLC38A9) in antiquewhite4 module, Interferon Induced With Helicase C Domain 1 (IFIH1), Interferon‐Induced Protein With Tetratricopeptide Repeats 2 (IFIT2), Interferon‐Induced Protein With Tetratricopeptide Repeats 3 (IFIT3), 2′‐5’‐Oligoadenylate Synthetase 1 (OAS1), 2′‐5’‐Oligoadenylate Synthetase 2 (OAS2), XIAP Associated Factor 1 (XAF1) and Radical S‐Adenosyl Methionine Domain Containing 2 (RSAD2) in the brown module, and GINS Complex Subunit 2 (GINS2), Aurora Kinase B (AURKB), and Cell Division Cycle 45 (CDC45) in darkmagenta module, as hub genes of PPI, were identified by the cytoHubba.

### 3.6 miRNA‐lncRNA‐mRNA‐TF co‐expression network

**FIGURE 7:**
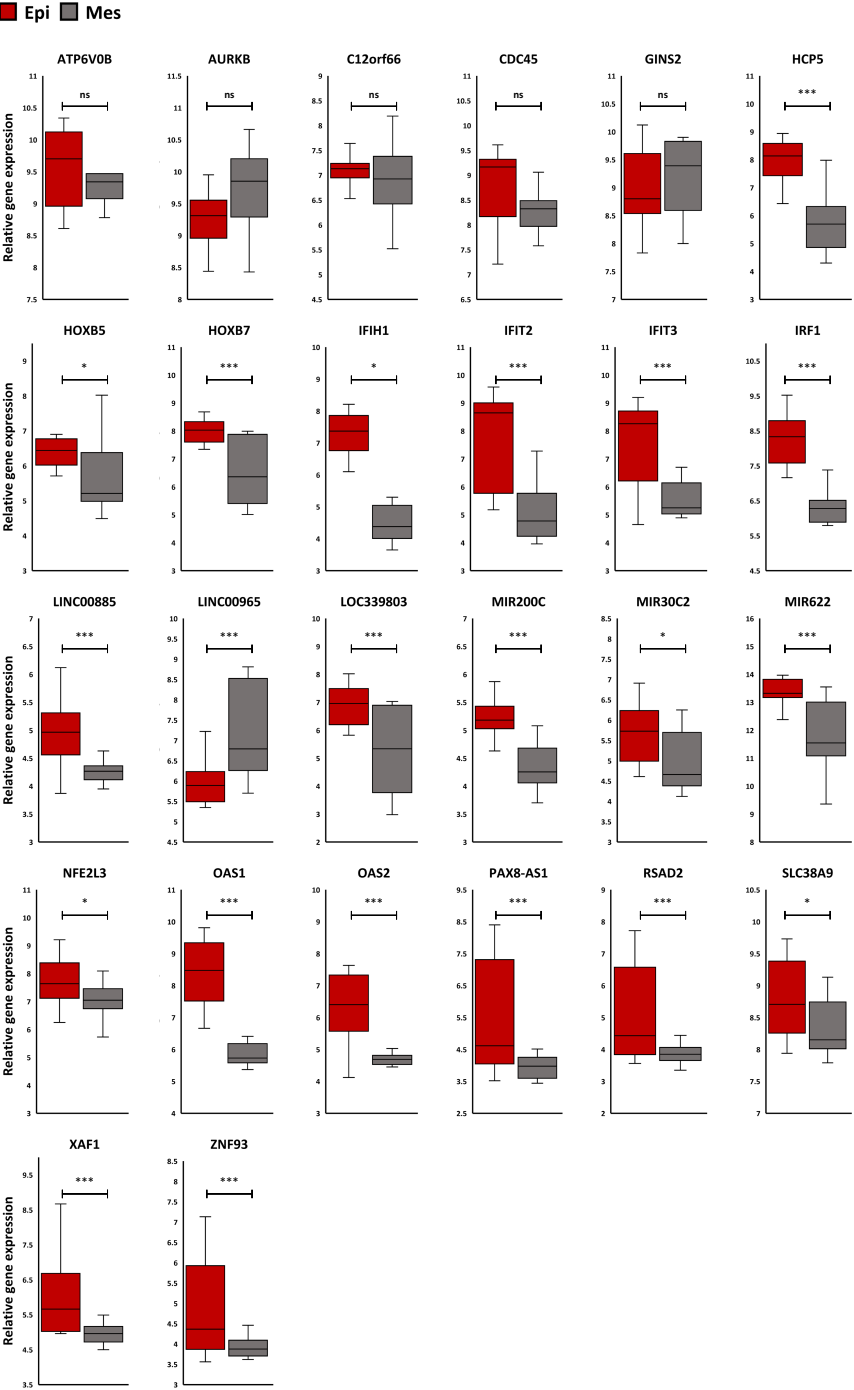
The relative expression of each module member between epithelial and mesenchymal groups. Expression boxplot of hub genes and their co‐expressed miRNA‐lncRNA‐TF with hub genes among epithelial and mesenchymal ovarian cancer cell lines. Indicated *p*‐value as less than 0.05, 0.01 and 0.001 illustrated with one, two and three stars, respectively

The miRNAs, lncRNAs and TFs co‐expressed with hub genes in each module were filtered out based on (|log2FC| ≥ 0.58 and adjusted *p*‐value <0.05). The antiquewhite4 module contains one miRNA, miR‐30C2 and four functional TFs, including Homeobox B5 (HOXB5), Homeobox B7 (HOXB7), Zinc Finger Protein 93 (ZNF93) and NFE2 Like BZIP TF3 (NFE2L3) (Figure 6B). The brown module contains two miRNAs, one functional TF and one lncRNA, including miR‐200c, miR‐622, Interferon Regulatory Factor 1 (IRF1) and HLA Complex P5 (HCP5), respectively (Figure 6D). The darkmagenta module contains four lncRNAs, including PAX8 Antisense RNA 1 (PAX8‐AS1), LOC339803, Long Intergenic Non‐Protein Coding RNA 965 (LINC00965) and Long Intergenic Non‐Protein Coding RNA 885 (LINC00885) (Figure 6F). The relative expression of each module member is shown in Figure 7. To identify the regulation of each module through circular RNAs (circRNAs) literature review was used to discover circRNAs, including experimentally validated interactions with miRNAs in candidate modules. Circ_VMA21 and circ_0057481 may regulate the brown module through miR‐200c and circ_GLG1, circ_TMX4, circ_MTUS1, circ_UBXN7, circ_0119872, circ_0000231 and circ_0000211 may regulate this module through miR‐622.

### 3.7 The hub genes were validated using other datasets

**FIGURE 8:**
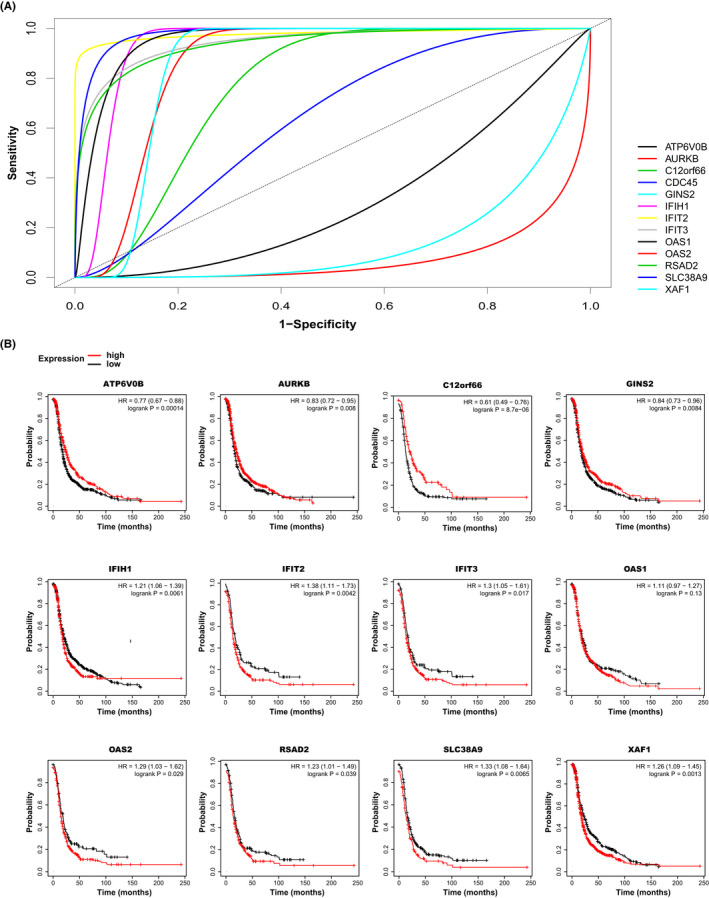
Hub genes were validated using ROC, Kaplan–Meier plot. (A) ROC plot of candidate genes to validate their discrimination ability by another dataset. (B) Kaplan–Meier curves to survival analysis of candidate genes regarding platinum‐treated patients through TCGA and GEO. Roles of the up‐regulated key genes in Epithelial cisplatin resistance associated with a lower overall survival rate imply cancer invasion after treatment

Data validation was accomplished using the GSE149146 dataset, including three platinum‐sensitive and three platinum‐resistant cell lines. The PCA analysis was performed to distinguish and remove outlier samples from the dataset. Subsequently, ROC curve analysis was used to evaluate the diagnostic prediction values of the hub genes as candidate prognostic biomarkers of cisplatin resistance (Figure 8A). Our results show that AUC for OAS1 was 0.9519 (*p* < 0.05). At the optimal cut‐off value of 3.85, both sensitivity and specificity were 100%. Similar results were obtained for SLC38A9, OAS2, IFIT2, IFIT3, IFIH1, RSAD2 and XAF1 (Figure 8A; Table 1). These results demonstrate that these hub genes possessed a high ability to discriminate between cisplatin resistance and sensitivity. To analyse the expression signature of candidate hub genes as prognostic biomarkers in response to platinum treatment, publicly available data and tools from GEO, EGA and TCGA databases were utilized. As shown in Figure 8B, the Kaplan–Meier survival analysis results show the high expression levels of IFIH1, IFIT2, IFIT3, OAS2, RSAD2 SLC38A9 and XAF1 are the risk factors affecting the prognosis of ovarian platinum‐based treated patients (*p* < 0.05). However, the patients with lower ATP6V0B, AURKB, C12orf66 and GINS2 genes expression levels showed lower overall survival rates. In contrast, the expression of the CDC45 gene did not significantly affect the patient's prognosis (Figure 8B).

**TABLE 1:** The results of AUC analysis for candidate hub genes in candidate modules

Module	Marker	AUC	*p*‐Value	LowerLimit	UpperLimit	Sensitivity	Specificity
Brown	RSAD2	0.9377362	1.19E‐10	0.804535	1	1	0.83
	OAS1	0.9518966	8.01E‐04	0.6876886	1	1	1
	OAS2	0.8580055	1.23E‐02	0.5778157	1	0.667	1
	IFIT2	0.9775556	2.24E‐53	0.9166971	1	1	1
	IFIT3	0.9444248	4.21E‐12	0.8187296	1	1	0.833
	IFIH1	0.9328008	1.29E‐02	0.5916245	1	1	1
	XAF1	0.8543789	1.88E‐02	0.5587648	1	1	0.83
Antiquewhite4	ATP6V0B	0.3098784	2.05E‐01	0.01566976	0.6040871	1	0
	SLC38A9	0.9777828	4.34E‐05	0.7487513	1	1	0.833
	C12orf66	0.766804	5.79E‐02	0.49102256	1	1	0.5
Darkmagenta	AURKB	0.07941252	0.01046927	0	0.4014275	1	0
	CDC45	0.6341437	0.3901268	0.3282086	0.9400789	1	0.5
	GINS2	0.14384058	0.01511212	0	0.4311456	1	0

## 4 DISCUSSION

Platinum resistance hinders the development of effective ovarian cancer therapeutics. Many studies show the importance of EMT in disease processes such as tumour progression. Emerging evidence indicates a role for EMT in the drug resistance development in cancer cells, like resistance to paclitaxel in breast cancer cell lines with the transitioned phenotype.^12^, ^13^, ^15^, ^17^ In vitro treatment of breast, colorectal and ovarian cancer cells with chemotherapeutic agents like cisplatin, oxaliplatin, doxorubicin and paclitaxel results in drug resistance. It has been shown that this drug resistance is associated with developing a transitioned phenotype.^16^, ^18^, ^22^ Cisplatin‐treated cancer cell lines acquired cisplatin resistance accompanied by NF‐κB activation and impairment of apoptosis.^7^ However, there is a lack of precise therapeutic and prognostic biomarkers related to EMT phenotype and drug resistance in ovarian cancer. Therefore, it is essential to prospect genes associated with drug resistance in this cancer.

Using bioinformatics analysis, our research identifies critical genes that correlate strongly with EMT phenotype and chemotherapeutic resistance in this cancer. The most widely used co‐expression network method, WGCNA, has been used in various applications, like cancer genetic analysis. WGCNA is notably useful for identifying the gene co‐expression modules that correlate with tumour clinical traits and biological behaviour. WGCNA has many advantages over other differential expression analysis methods since its focus is on co‐expression patterns, which helps discover functional modules containing related genes. Hub genes in the non‐preserved modules related to particular traits may be used as prognosis or diagnosis biomarkers or therapeutic targets.

In this study, 3201 DEGs comprising 1524 up‐regulated and 1677 down‐regulated genes with |log2FC| ≥ 0.58 and adjusted *p*‐value <0.05 were discerned from the dataset. We employed WGCNA to investigate gene co‐expression alternation between ovarian cancer cell lines with an epithelial phenotype and those with a mesenchymal phenotype. Using module preservation analysis, we found that four modules (Brown, Antiquewhite4, Darkmagenta and Darkturquoise) were significantly related to the epithelial phenotype, which indicated the implication of these modules in the progress of cisplatin resistance in ovarian cancer cell lines. Then, Venn diagrams were represented to show the overlap between the DEGs and genes in non‐preserved modules. The brown, antiquewhite4 and darkmagenta modules have the most overlap with up and down‐regulated genes.

Further GO functional enrichment analysis showed that the brown module was primarily enriched in the extracellular matrix and external encapsulating structure organization, cell morphogenesis, amoeboidal‐type and epithelial cell migration processes, and focal‐adhesion and ECM receptor interaction pathways. The ECM receptor interactions were shown to induce cell adhesion‐mediated drug resistance (CAM‐DR) in EOC.^48^ Januchowski et al.^49^ reported that the extracellular matrix (ECM) could affect drug resistance progression in ovarian cancer by prohibiting the drug penetration into cancer cells and developing resistance to apoptosis. The enrichment analysis of the antiquewhite4 module genes suggests the role of cellular stress response and detoxifying processes and proton and ions transporting ATPase activity functions in the progression of drug resistance in epithelial ovarian cancer. A common feature among drug‐resistant cells is the upregulation of the ABC transporters, active in multi‐drug resistance pathways. It efficiently removes hydrophobic molecules, like taxol and doxorubicin, from the cell.^50^ Besides, GO and KEGG analysis of the darkmagenta module explicit the enrichment of the DNA replication processes in this module. Platinum‐based drugs induce a state of replication stress and severely block the progression of DNA polymerases. It selectively promotes the elimination of cancer cells proliferation.^51^ Specifically, a majority of ovarian cancer cell lines exhibit strong defects in nucleotide excision repair (NER) during the S phase relative to G0–G1 and G2–M after platinum‐based treatment.^52^ Hence, the generation of ssDNA at RNA polymerase stalling sites at platin‐damaged DNA sites might promote replication stress via collisions between the blocked transcriptional machinery and DNA polymerases.^53^ Although the precise mechanisms remain incompletely known for removing DNA lesions or inhibiting their formation, modulation of the cellular response to replication stress, like pathways that stabilize or resolve stalled replication forks, is a key determinant of cisplatin resistance in cancer.^54^

Next, the STRING database was used to construct the PPI network of genes belonging to the candidate modules. CytoHubba was used to identify the hub genes of the top‐scored MCODE cluster in each module based on the MCC score. The brown module contains the top seven hub genes, including RSAD2, OAS1, OAS2, IFIT2, IFIT3, IFIH1 and XAF1. The expression levels of these hub genes were evaluated in ovarian cancer cell lines with epithelial phenotype and mesenchymal phenotype. Survival analysis indicates the potential prognostic value of these hub genes in ovarian cancer. In this module, one TF (IRF1), one lncRNA (HCP5) and two miRNAs (miR‐200C and miR‐622) were significantly co‐expressed with the identified hub genes. Literature review reveals that nine circRNAs may regulate this coexpression network through miRNA‐200c and miR‐622 including circ_VMA21, circ_0057481, circ_GLG1, circ_TMX4, circ_MTUS1, circ_UBXN7, circ_0119872, circ_0000231 and circ_0000211.^55^, ^56^, ^57^, ^58^, ^59^, ^60^, ^61^, ^62^, ^63^

Studies show that RSAD2 is up‐regulated and associated with worse relapse‐free survival in breast cancer.^64^ Tang et al.^65^ reported that the overexpression of this immune‐related gene is associated with tumour grade, stage and size in breast cancer. Albeit, some studies have shown the anti‐tumour role of RSAD2 in lung and oral cancers.^66^, ^67^ Our analysis indicates that RSAD2 is up‐regulated in epithelial ovarian cell lines, and this overexpression is significantly associated with poor prognosis. It has been shown that OAS family members are involved in a variety of diseases, like autoimmune disorders, infections and cancer, as the regulator of cell growth, differentiation, gene regulation and apoptosis.^68^, ^69^, ^70^, ^71^ Researchers indicate that the activity of this family is related to cancer progression. For example, OAS1 activates cell migration.^72^ Moreover, a recent study revealed that high expression of OAS1 predicts poor survival in breast cancer patients.^73^ Multiple stimuli can induce IFITs, such as interferon (IFN)‐dependent or IFN‐independent signalling pathways. Based on stimulus, cell type and tissue type, IFITs can execute numerous complex functions.^74^ Studies demonstrated a correlation between IFIT2 down‐regulation and EMT induction, metastasis and multi‐drug resistance in patients with oral squamous cell carcinoma (OSCC). IFIT2 is significantly overexpressed in ovarian cancer stem cells, indicating its potential role in drug resistance in ovarian cancer.^75^ Nonetheless, elevated IFIT3 expression correlates with poor survival and increased resistance to various chemotherapeutic drugs, including cisplatin in OSCC patients.^74^ IFIT proteins regulate multiple complex cellular processes based on cell and tissue types. Therefore, depending on the cell system, its functions could be altered.

The IFIH1 gene encodes the MDA5 (Melanoma Differentiation‐Associated protein 5) protein, which has a role in the antiviral response by recognizing dsRNA.^76^ After dsRNA recognition, IFIH1 activates a signalling cascade that induces the type I interferon and interferon‐stimulated genes that initiate antiviral immune responses. Studies show down‐regulation of the IFIH1 gene is associated with Docetaxel resistance in prostate cancers.^77^ On the other hand, the overexpression of IFIH1 is involved in ovarian cancer drug resistance, indicating the role of IFIH1 is cancer‐dependent.^78^ The expression of XAF1, a pro‐apoptotic protein, is frequently suppressed in human cancers. Studies show a positive feedback loop between XAF1 and p53 by direct binding to the N‐terminal domain of p53, which supports the tumour suppressor role of XAF1 in a p53‐dependant manner.^79^, ^80^, ^81^ XAF1 expression is reduced in various cancers, including ovarian.^82^ Pieces of evidence show that overexpression of XAF1 increased the cisplatin sensitivity of SKOV3 cell lines, which have a mesenchymal phenotype.^7^, ^82^, ^83^ Nevertheless, some evidence shows the over‐expression of XAF1 promotes Temozolomide resistance in GBM cell lines and is negatively correlated with long‐term survival in glioblastoma patients.^84^ Over‐expression of XAF1F, one of XAF1 transcript variants, in gastric cancer‐derived circulating tumour cells with EMT characteristics is shown to promote tumour invasion, lymph node metastasis and venous invasion.^85^ However, our study shows the overexpression of XAF1 is positively correlated to epithelial phenotype and cisplatin resistance in ovarian cancer cell lines, indicating the phenotype‐dependent role of XAF1 in ovarian cancer cisplatin resistance. Some studies show the association of IRF1 overexpression with increased overall survival of ovarian carcinoma patients.^86^ However, IRF1 silencing improved Taxol sensitivity in ovarian cancer cells.^87^ Cisplatin is more effective in proliferating cells. Pavan et al.^88^ discovered that IRF1 expression in ovarian cancer cell lines is elevated in response to cisplatin treatment, limiting the cell response to cisplatin through cell cycle arrest at G1 phase. It has been shown that lncRNA HCP5 plays a cancer‐promoting role in several cancer types, such as pancreatic, colorectal, lung and thyroid cancers.^89^ It was first observed that HCP5 was significantly downregulated in ovarian cancer.^90^ However, the latest study by Wang et al.^91^ revealed that the upregulation of HCP5 in ovarian cancer tissues and cells induced the EMT process via the HCP5/miR‐525‐5p/PRC1 axis and increased the proliferation, invasion and migration of these cells. Recent studies show HCP5 promotes proliferation and contributes to cisplatin resistance in gastric cancer cells.^92^, ^93^ Overexpression of tumour miR‐622 was also associated with a poor prognosis in ovarian cancer patients.^94^ Choi YE et al.^95^ reported that miR‐622 leads to platinum resistance in ovarian cancer cell lines. miR‐200c is a member of the miR‐200 family. This family has been associated with the cancer stem cells formation and regulation of the EMT process.^96^, ^97^ Overexpression of miR‐200c induces cisplatin resistance in the A2780 ovarian cancer cell line.^98^ However, other studies show the overexpression of miR‐200c increases the cisplatin sensitivity of ovarian cancer cells.^99^ Our analysis indicates that the over‐expression of miR‐141 and miR‐200c can cause cisplatin resistance through the EMT process. These findings suggest that more experimental proof on different ovarian cancer cell lines is needed to confirm the role of these miRNAs in ovarian cancer cisplatin resistance.

The antiquewhite4 module contains three hub genes, including ATP6V0B, SLC38A9 and C12orf66. These hub genes were not significantly dysregulated in epithelial compared to mesenchymal phenotype. In this module, four TFs (HOXB5, HOXB7, NFE2L3 and ZNF93) and one miRNA (miR‐30C‐2) were co‐expressed with the identified hub genes. Vacuolar ATPase (V‐ATPase), a multisubunit enzyme, has an integral V0 subunit that mediates the acidification of eukaryotic intracellular organelles.^100^ A higher expression level of ATP6V0B has been reported in oesophageal squamous cell carcinoma (ESCC) and metastatic melanoma samples.^101^, ^102^ SLC38A9 promotes cancer cell proliferation and tumour growth by activating mTORC1, which is commonly activated in tumours and supports biosynthetic needs for the rapid proliferation of cancer cells.^103^ This gene was shown to be down‐regulated in drug‐resistant ovarian cancer cell lines.^50^ There are not enough validated studies discovering the role of C12orf66 in cancer progression and drug resistance. The solid experimental proof is required to illustrate the precise role of this gene in cancer progression. Highly conserved members of the homeobox superfamily (HOX) encode a TF with a fundamental role in regulating various cellular mechanisms.^104^ The growing evidence mentions that they have a fundamental oncogenesis role in several tumour types, including colorectal, breast and ovarian cancer.^104^, ^105^ HOXB5 and HOXB7 are significantly up‐regulated in serous epithelial ovarian cancers.^106^ Evidence shows the overexpression of HOXB7 promotes cell survival and induces chemo‐radiotherapy resistance in oral cancer.^107^ NRF3 promotes tumour suppressor p53 degradation, which increases cancer cell proliferation.^34^ The overexpression of NFE2L3 promotes proliferation and metastasis in hepatocellular carcinoma and colon cancer.^108^, ^109^ Evidence shows that ZNF93 upregulation in cisplatin‐resistant ovarian cancer cell lines is associated with low survival, and its knockdown enhanced sensitivity to cisplatin.^110^ The miR‐30c‐2, a member of the miR‐30 family, is generally recognized as a multifunctional regulator of cell proliferation, differentiation, metabolism and apoptosis, which is related to cancer metastasis and chemoresistance in‐vivo. Jia et al.^111^ reported that the overexpression of miR‐30c‐2 in the ovarian cell proliferation process could induce cell proliferation during ovarian cancer progression. Our analysis indicates the upregulation of miR‐30c‐2 in epithelial ovarian cell lines may be related to cisplatin resistance status.

The darkmagenta module includes three hub genes, including AURKB, CDC45 and GINS2. Like antiquewhite4 hub genes, these genes show no differential expression between the Epithelial and Mesenchymal phenotypes. These hub genes are co‐expressed with four lncRNAs (LOC339803, LINC00965, LINC00885 and PAX8‐AS1). The overexpression of AURKB has been shown in various human cancers, including ovarian cancer.^112^ This overexpression is associated with worse overall survival in ovarian cancer.^113^ Studies show the association of AURKB overexpression with cisplatin resistance in gastric cancer cells, tamoxifen resistance in breast cancer and cetuximab resistance in head and neck squamous cell carcinoma.^114^, ^115^, ^116^ CDC45, a proliferation‐associated antigen, is up‐regulated in human cancer cell lines promoting cancer cell division.^117^ Studies show that CDC45 overexpression is involved in colorectal, papillary thyroid and non‐small cell lung cancer initiation and progression.^118^, ^119^, ^120^ A recent study shows that CDC45 knockdown impaired DNA damage and induced resistance to combination therapy in ovarian cancer cells.^121^ Evidence shows that GINS2 (GINS complex subunit 2) has a role in regulating cell cycle and apoptosis and is overexpressed in different malignant cancers, including pancreatic, lung and thyroid cancers.^122^, ^123^, ^124^ GINS2 is overexpressed in human EOC, promotes cancer progression and impairs apoptosis.^125^ LOC339803, a newly discovered lncRNA, was shown to have a role in the progression of hepatocellular carcinoma cells (HCC). The overexpression of LOC339803 in HCC is associated with HCC invasion and lower survival time.^126^ Our results show that the overexpression of this lncRNA is also associated with a lower survival rate in ovarian cancer. LINC00965 is a newly discovered lncRNA, and there is no data about it. Abba et al. reported the molecular effects of LINC00885 as a new oncogenic lncRNA associated with early breast cancer progression. Overexpression of this lncRNA is closely associated with a lower survival rate and poor prognosis in breast and cervical cancer patients.^127^, ^128^ Lu et al.^129^ demonstrated that high expression of PAX8‐AS1 is associated with poor relapse‐free survival in thyroid cancer patients. However, a recent study reported the tumour suppressor role of PAX8‐AS1.^130^ PAX8 has a role in transforming the ovarian cancer cell into mesenchymal phenotype.^131^ We hypothesize that PAX8‐AS1 may maintain epithelial characteristics in these cells by regulating PAX8.

In summary, we provide a Holistic biological interpretation of gene expression data derived from different ovarian cancer cell lines. WGCNA analysis identified 40 modules, 4 of which were significantly associated with an epithelial phenotype. We suggest that the brown module contains a potential prognostic biomarker of ovarian cancer progression and drug resistance based on the expression pattern, ROC curve and survival analysis. This module includes 10 hub/key genes co‐expressed with different TFs, miRNAs, and lncRNAs. Pathway analysis implicates that this co‐expression pattern may have a role in developing drug resistance in ovarian cancer. This study also provides several circRNAs for future in vitro and in vivo investigations of their molecular mechanisms regulating cisplatin resistance in ovarian cancer. Our findings add to our understanding of the processes and genes that underpin ovarian drug resistance through the EMT process. However, solid experimental proof is required to confirm our predictions.

## AUTHOR CONTRIBUTIONS

**Amirhosein Naghsh‐Nilchi:** Investigation (equal); methodology (equal); writing – original draft (equal). **Laleh Ebrahimi Ghahnavieh:** Investigation (equal); methodology (equal); writing – original draft (equal). **Fariba Dehghanian:** Investigation (lead); methodology (supporting); project administration (lead); validation (lead); writing – review and editing (lead).

## CONFLICT OF INTEREST

The authors declare that the research was conducted without any commercial or financial relationships that could be construed as a potential conflict of interest.

## Supporting information


Appendix S1

## Data Availability

The data that support the findings of this study are openly available in the NCBI Gene Expression Omnibus (GEO) at (https://www.ncbi.nlm.nih.gov/geo/), reference number (GSE47856, GSE149146).
