# Supplementary material for: Construction of miRNA‐lncRNA‐mRNA co‐expression network affecting EMT‐mediated cisplatin resistance in ovarian cancer
Source: J Cell Mol Med. 2022 Jul 10;26(16):4530–47. doi: 10.1111/jcmm.17477 (PMC9357632; doi:10.1111/jcmm.17477)
Supplement: Supplementary file 1 — Appendix S1 [file JCMM-26-4530-s001.docx]

**Appendices**


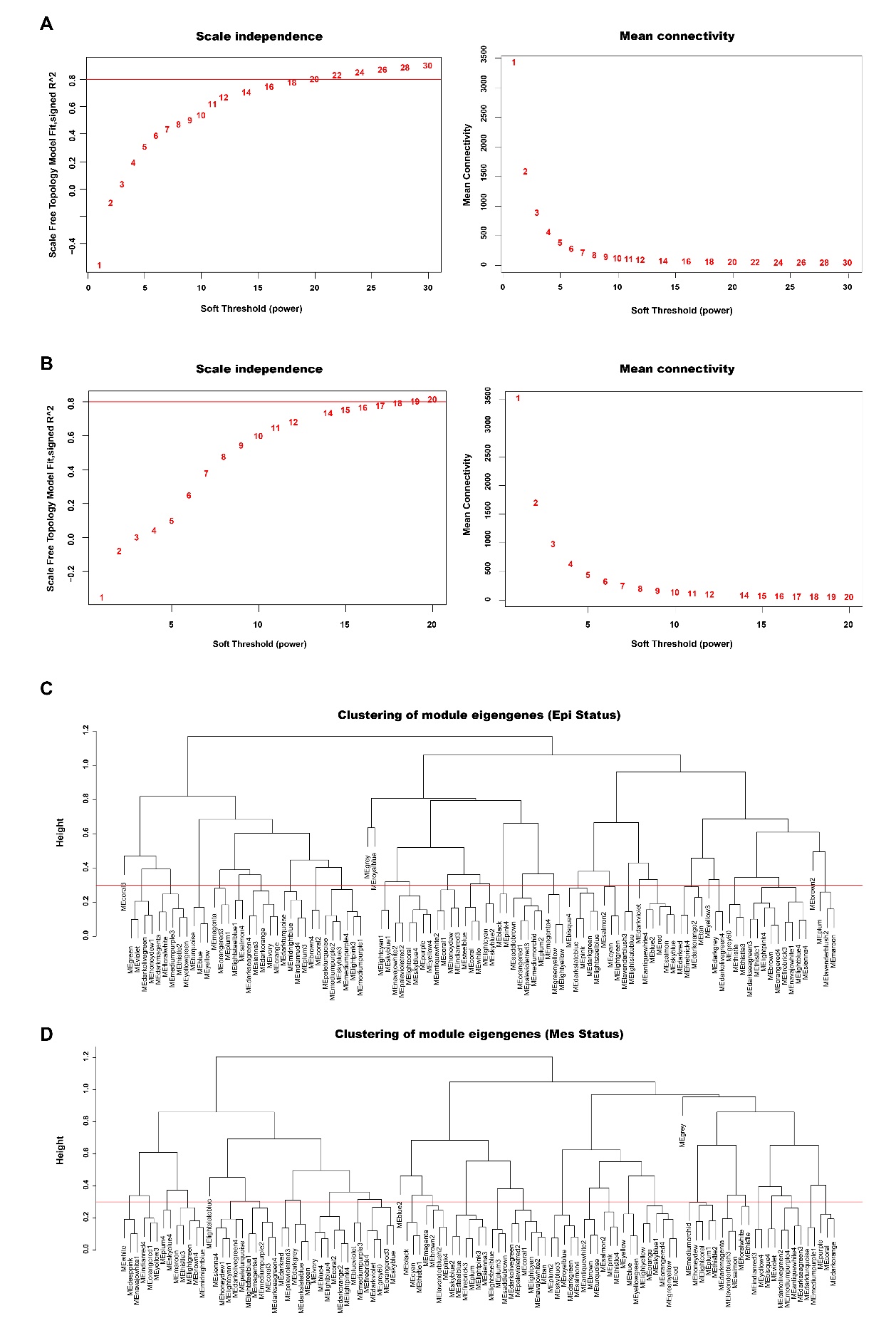


**Supplementary Figure 1.** **The WGCNA network and the gene co-expression modules were constructed using hierarchical clustering for module detection.** (**A/B**) Selection of the soft-thresholding powers for Mesenchymal and Epithelial cell lines. The left panels showed the scale-free fit index versus soft-thresholding power. The right panels displayed the mean connectivity versus soft-thresholding power. The chosen power for both Mesenchymal and Epithelial samples is 9. (**C/D**) Hierarchical clustering dendrograms of module eigengenes in (**C**) Epithelial and (**D**) Mesenchymal modules based on correlated modules. Several modules were generated, and MEDissThres, 0.3 for both groups, was used to merge the clustered gene modules.


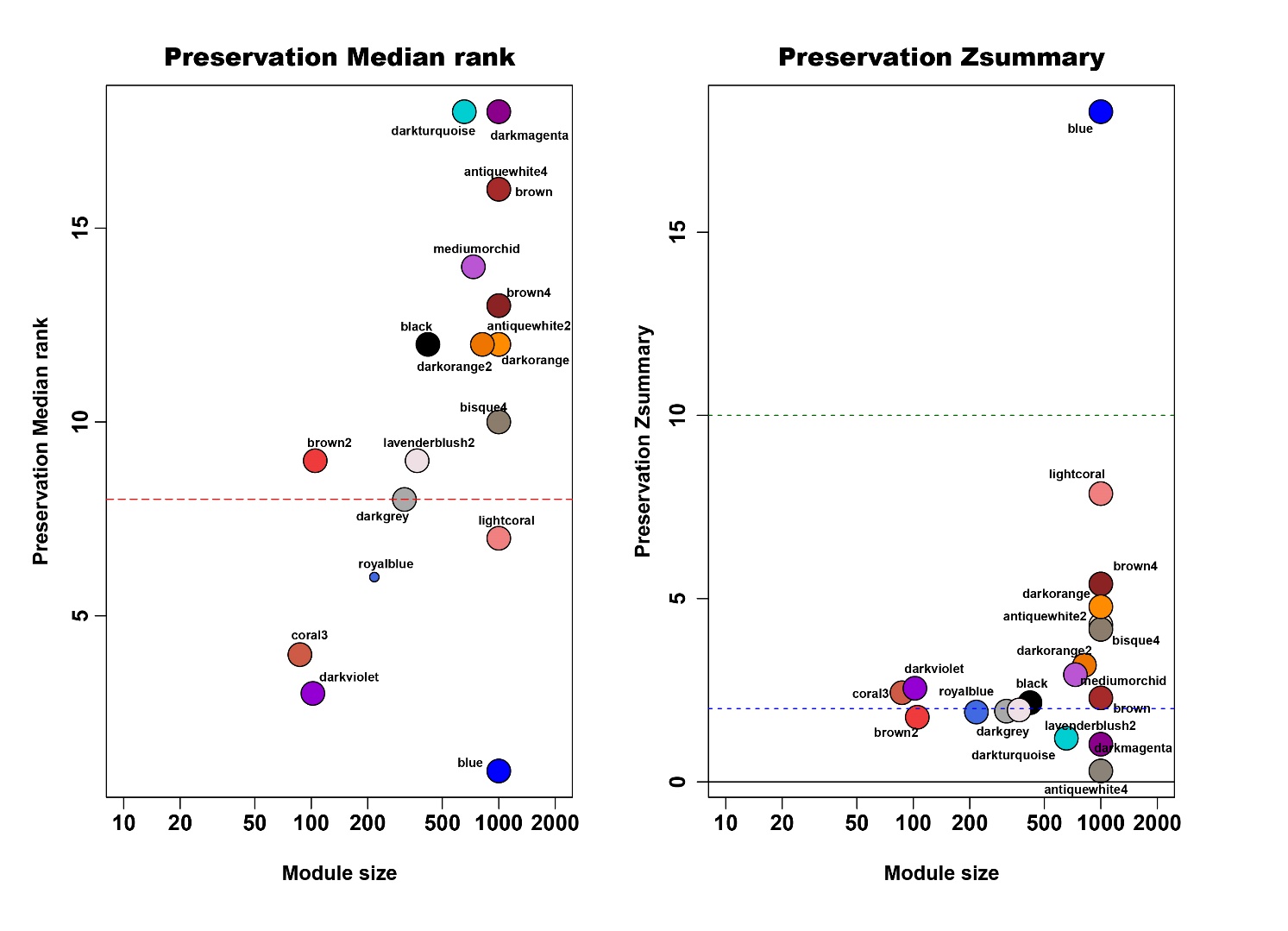


**Supplementary Figure 2.** **Evaluation of module preservation by median rank and Zsummary statistics.** The plots show the module position in the Epithelial status based on the medianRank (left) and the Zsummary (right). Each point shows a color-coded module. The dashed blue and green lines in the preservation Zsummary plot indicate the threshold Z = 2 & Z = 10. The dashed red line in the preservation medianRank plot indicates the threshold mR = 8.


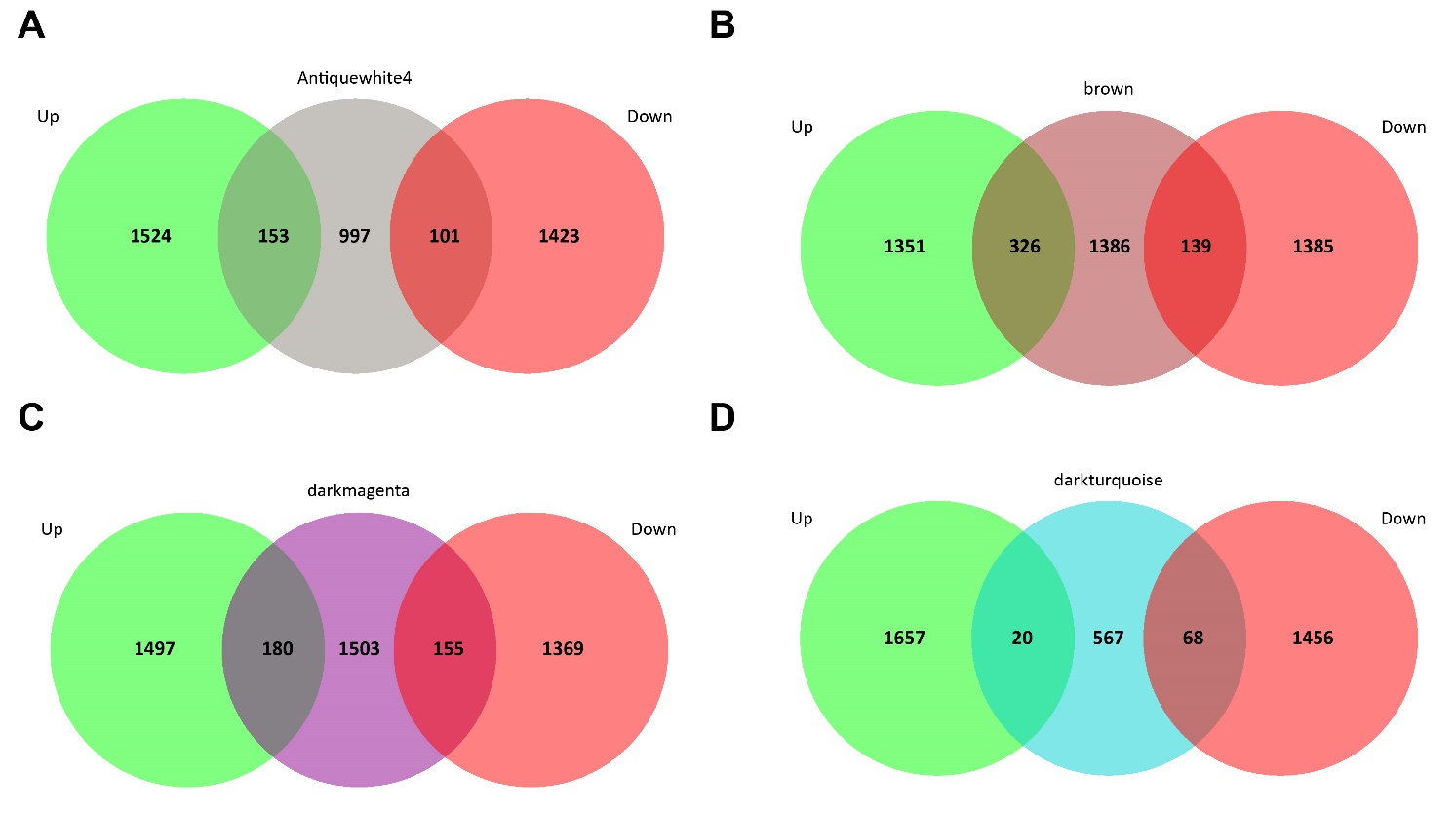


**Supplementary Figure 3.** **Overlap of DEGs and non-preserved modules separately.** Venn diagram of non-preserved modules, up (green) and down (red) regulated genes. The numbers inside the diagram represent the overlapped genes shared between different categories of DEG and modules. (**A**) Genes of the Antiquewhite4 module (**B**) Genes of the Brown module, (**C**) Genes of the Darkmagenta module, (**D**) Genes of the Darkturquoise module.

**Supplementary Table 1.** The number of genes per module (module size) ranging in Mesenchymal and Epithelial.

| **Mesenchymal** | | **Epithelial** | |
| --- | --- | --- | --- |
| **Module** | **Genes** | **Module** | **Genes** |
| antiquewhite2 | 4065 | darkmagenta | 1838 |
| blueviolet | 1910 | blue | 4637 |
| coral1 | 1598 | brown | 1851 |
| coral3 | 1436 | darkorange | 1849 |
| greenyellow | 1376 | brown4 | 1626 |
| black | 1352 | bisque4 | 1515 |
| darkgrey | 1140 | antiquewhite4 | 1251 |
| brown4 | 1084 | antiquewhite2 | 1246 |
| antiquewhite4 | 1016 | lightcoral | 1142 |
| darkgreen | 961 | darkorange2 | 818 |
| darkmagenta | 895 | mediumorchid | 732 |
| firebrick3 | 867 | darkturquoise | 655 |
| honeydew | 743 | black | 419 |
| deeppink | 715 | lavenderblush2 | 367 |
| coral | 606 | darkgrey | 314 |
| pink | 493 | royalblue | 217 |
| darkturquoise | 307 | brown2 | 105 |
| blue2 | 134 | darkviolet | 102 |
| lightslateblue | 85 | coral3 | 87 |
| grey | 66 | grey | 78 |

**Supplementary Table 2.** The preservation statistics of Epithelial modules with the number of genes per module (module size).

| **Module_Name** | **MedianRank** | **Zsummary** | **Module_Size** | **Preservation_Type** |
| --- | --- | --- | --- | --- |
| darkmagenta | 18 | 1 | 1838 | Non-preserved |
| darkturquoise | 18 | 1.2 | 655 | Non-preserved |
| antiquewhite4 | 16 | 0.3 | 1251 | Non-preserved |
| brown | 16 | 2.3 | 1851 | Non-preserved |
| mediumorchid | 14 | 2.9 | 732 | Non-preserved |
| brown4 | 13 | 5.4 | 1626 | Non-preserved |
| antiquewhite2 | 12 | 4.3 | 1246 | Non-preserved |
| black | 12 | 2.2 | 419 | Non-preserved |
| darkorange | 12 | 4.8 | 1849 | Non-preserved |
| darkorange2 | 12 | 3.2 | 818 | Non-preserved |
| bisque4 | 10 | 4.2 | 1515 | Non-preserved |
| brown2 | 9 | 1.8 | 105 | Non-preserved |
| lavenderblush2 | 9 | 2 | 367 | Non-preserved |
| darkgrey | 8 | 1.9 | 314 | Non-preserved |
| lightcoral | 7 | 7.9 | 1142 | Preserved |
| royalblue | 6 | 1.9 | 217 | Non-preserved |
| coral3 | 4 | 2.4 | 87 | Non-preserved |
| darkviolet | 3 | 2.6 | 102 | Non-preserved |
| blue | 1 | 18 | 4637 | Preserved |

**Supplementary Table 3.** The topological parameters of top MCODE Cluster in candidate modules.

| **Module** | **Symbol** | **MCC** | **Degree** | **EcCentricity** | **Closeness** | **Radiality** | **Betweenness** | **Stress** |
| --- | --- | --- | --- | --- | --- | --- | --- | --- |
| **Antiquewhite4** | **ATP6V0B** | 7257600 | 11 | 1 | 11 | 2.27273 | 0.2 | 2 |
|  | **ATP6V0E1** | 7257600 | 11 | 1 | 11 | 2.27273 | 0.2 | 2 |
|  | **ATP6V1B2** | 7257600 | 11 | 1 | 11 | 2.27273 | 0.2 | 2 |
|  | **ATP6V1D** | 7257600 | 11 | 1 | 11 | 2.27273 | 0.2 | 2 |
|  | **ATP6V1F** | 7257600 | 11 | 1 | 11 | 2.27273 | 0.2 | 2 |
|  | **ATP6V1G3** | 7257600 | 11 | 1 | 11 | 2.27273 | 0.2 | 2 |
|  | **C12orf66** | 7257600 | 11 | 1 | 11 | 2.27273 | 0.2 | 2 |
|  | **C7orf60** | 3628800 | 10 | 0.5 | 10.5 | 2.18182 | 0 | 0 |
|  | **FNIP1** | 3628800 | 10 | 0.5 | 10.5 | 2.18182 | 0 | 0 |
|  | **LAMTOR5** | 7257600 | 11 | 1 | 11 | 2.27273 | 0.2 | 2 |
|  | **RHEB** | 7257600 | 11 | 1 | 11 | 2.27273 | 0.2 | 2 |
|  | **SLC38A9** | 7257600 | 11 | 1 | 11 | 2.27273 | 0.2 | 2 |
| **Brown** | **ADAR** | 2.49E+10 | 16 | 0.5 | 19.5 | 1.82609 | 0.92403 | 12 |
|  | **EPSTI1** | 1.87E+10 | 15 | 0.5 | 19 | 1.78261 | 0.40952 | 6 |
|  | **GBP1** | 3.08E+12 | 21 | 0.5 | 22 | 2.04348 | 3.651 | 48 |
|  | **HLA-F** | 6.23E+09 | 13 | 0.5 | 18 | 1.69565 | 0 | 0 |
|  | **IFI27** | 2.65E+12 | 20 | 0.5 | 21.5 | 2 | 3.38649 | 42 |
|  | **IFI44L** | 1.78E+12 | 21 | 0.5 | 22 | 2.04348 | 3.91381 | 50 |
|  | **IFI6** | 2.75E+12 | 21 | 0.5 | 22 | 2.04348 | 4.38453 | 54 |
|  | **IFIH1** | 3.09E+12 | 22 | 0.5 | 22.5 | 2.08696 | 4.83368 | 62 |
|  | **IFIT2** | 3.10E+12 | 23 | 1 | 23 | 2.13043 | 6.78046 | 82 |
|  | **IFIT3** | 3.10E+12 | 23 | 1 | 23 | 2.13043 | 6.78046 | 82 |
|  | **IFNA1** | 1.25E+10 | 14 | 0.5 | 18.5 | 1.73913 | 0.13333 | 2 |
|  | **IRF1** | 2.82E+12 | 21 | 0.5 | 22 | 2.04348 | 4.39286 | 54 |
|  | **MX2** | 3.08E+12 | 22 | 0.5 | 22.5 | 2.08696 | 5.3909 | 66 |
|  | **OAS1** | 3.10E+12 | 23 | 1 | 23 | 2.13043 | 6.78046 | 82 |
|  | **OAS2** | 3.10E+12 | 23 | 1 | 23 | 2.13043 | 6.78046 | 82 |
|  | **PARP14** | 3.49E+11 | 16 | 0.5 | 19.5 | 1.82609 | 0.25833 | 4 |
|  | **PARP9** | 3.07E+12 | 20 | 0.5 | 21.5 | 2 | 2.28297 | 32 |
|  | **PSMB8** | 1.32E+12 | 17 | 0.5 | 20 | 1.86957 | 1.07289 | 14 |
|  | **RSAD2** | 3.10E+12 | 23 | 1 | 23 | 2.13043 | 6.78046 | 82 |
|  | **RTP4** | 3.07E+12 | 20 | 0.5 | 21.5 | 2 | 2.28297 | 32 |
|  | **SAMD9** | 1.81E+11 | 16 | 0.5 | 19.5 | 1.82609 | 0.55504 | 8 |
|  | **SAMD9L** | 2.74E+11 | 17 | 0.5 | 20 | 1.86957 | 0.83004 | 12 |
|  | **STAT2** | 2.91E+12 | 21 | 0.5 | 22 | 2.04348 | 4.16471 | 52 |
|  | **XAF1** | 3.09E+12 | 22 | 0.5 | 22.5 | 2.08696 | 5.23061 | 64 |
| **Darkmagenta** | **ASF1B** | 9.22E+13 | 48 | 0.5 | 48.5 | 2.04082 | 22.19494 | 500 |
|  | **ASPM** | 9.22E+13 | 48 | 0.5 | 48.5 | 2.04082 | 21.5607 | 492 |
|  | **AURKB** | 9.22E+13 | 49 | 1 | 49 | 2.06122 | 23.53047 | 532 |
|  | **CCNB2** | 9.22E+13 | 47 | 0.5 | 48 | 2.02041 | 19.83856 | 456 |
|  | **CDC25A** | 9.22E+13 | 29 | 0.5 | 39 | 1.65306 | 1.81242 | 46 |
|  | **CDC45** | 9.22E+13 | 49 | 1 | 49 | 2.06122 | 23.53047 | 532 |
|  | **CDCA3** | 9.22E+13 | 32 | 0.5 | 40.5 | 1.71429 | 4.56152 | 116 |
|  | **CDCA7** | 9.22E+13 | 38 | 0.5 | 43.5 | 1.83673 | 7.62533 | 186 |
|  | **CDT1** | 9.22E+13 | 46 | 0.5 | 47.5 | 2 | 19.16259 | 432 |
|  | **CENPM** | 9.22E+13 | 33 | 0.5 | 41 | 1.73469 | 4.63761 | 112 |
|  | **CENPN** | 9.22E+13 | 31 | 0.5 | 40 | 1.69388 | 2.30678 | 62 |
|  | **CHAF1A** | 9.22E+13 | 37 | 0.5 | 43 | 1.81633 | 7.90537 | 190 |
|  | **CHEK2** | 9.22E+13 | 28 | 0.5 | 38.5 | 1.63265 | 2.04848 | 52 |
|  | **CHTF18** | 9.22E+13 | 29 | 0.5 | 39 | 1.65306 | 3.38038 | 84 |
|  | **DLGAP5** | 9.22E+13 | 43 | 0.5 | 46 | 1.93878 | 12.62741 | 308 |
|  | **DTL** | 9.22E+13 | 43 | 0.5 | 46 | 1.93878 | 14.2985 | 334 |
|  | **DUT** | 9.22E+13 | 29 | 0.5 | 39 | 1.65306 | 3.34082 | 86 |
|  | **ESCO2** | 9.22E+13 | 33 | 0.5 | 41 | 1.73469 | 5.53055 | 134 |
|  | **FEN1** | 9.22E+13 | 46 | 0.5 | 47.5 | 2 | 17.96889 | 418 |
|  | **GINS2** | 9.22E+13 | 49 | 1 | 49 | 2.06122 | 23.53047 | 532 |
|  | **HELLS** | 9.22E+13 | 34 | 0.5 | 41.5 | 1.7551 | 4.45109 | 116 |
|  | **KIAA0101** | 9.22E+13 | 37 | 0.5 | 43 | 1.81633 | 6.56023 | 162 |
|  | **KIF11** | 9.22E+13 | 48 | 0.5 | 48.5 | 2.04082 | 21.5607 | 492 |
|  | **KIF22** | 9.22E+13 | 28 | 0.5 | 38.5 | 1.63265 | 3.8146 | 88 |
|  | **LIG1** | 9.22E+13 | 31 | 0.5 | 40 | 1.69388 | 3.91501 | 98 |
|  | **MCM2** | 9.22E+13 | 47 | 0.5 | 48 | 2.02041 | 20.1533 | 460 |
|  | **MCM6** | 9.22E+13 | 45 | 0.5 | 47 | 1.97959 | 16.60987 | 386 |
|  | **MKI67** | 9.22E+13 | 39 | 0.5 | 44 | 1.85714 | 8.42753 | 208 |
|  | **MYBL2** | 9.22E+13 | 31 | 0.5 | 40 | 1.69388 | 2.90174 | 74 |
|  | **NCAPD2** | 9.22E+13 | 38 | 0.5 | 43.5 | 1.83673 | 8.90511 | 210 |
|  | **NUSAP1** | 9.22E+13 | 43 | 0.5 | 46 | 1.93878 | 12.62741 | 308 |
|  | **ORC6** | 9.22E+13 | 29 | 0.5 | 39 | 1.65306 | 3.59878 | 88 |
|  | **PKMYT1** | 9.22E+13 | 32 | 0.5 | 40.5 | 1.71429 | 5.45413 | 122 |
|  | **PLK1** | 9.22E+13 | 46 | 0.5 | 47.5 | 2 | 18.44807 | 422 |
|  | **POLA1** | 9.22E+13 | 31 | 0.5 | 40 | 1.69388 | 2.99034 | 78 |
|  | **POLA2** | 9.22E+13 | 33 | 0.5 | 41 | 1.73469 | 5.32109 | 128 |
|  | **POLE2** | 9.22E+13 | 39 | 0.5 | 44 | 1.85714 | 9.62419 | 228 |
|  | **PRIM1** | 9.22E+13 | 42 | 0.5 | 45.5 | 1.91837 | 12.49192 | 294 |
|  | **RAD51** | 9.22E+13 | 45 | 0.5 | 47 | 1.97959 | 16.27893 | 380 |
|  | **RMI2** | 9.22E+13 | 25 | 0.5 | 37 | 1.57143 | 0.19655 | 6 |
|  | **RNASEH2A** | 9.22E+13 | 30 | 0.5 | 39.5 | 1.67347 | 3.51319 | 86 |
|  | **RRM1** | 9.22E+13 | 39 | 0.5 | 44 | 1.85714 | 8.66966 | 214 |
|  | **SHCBP1** | 9.22E+13 | 30 | 0.5 | 39.5 | 1.67347 | 4.50062 | 108 |
|  | **TOP2A** | 9.22E+13 | 48 | 0.5 | 48.5 | 2.04082 | 21.74003 | 492 |
|  | **TPX2** | 9.22E+13 | 43 | 0.5 | 46 | 1.93878 | 13.01069 | 312 |
|  | **TROAP** | 9.22E+13 | 28 | 0.5 | 38.5 | 1.63265 | 1.77245 | 46 |
|  | **TYMS** | 9.22E+13 | 46 | 0.5 | 47.5 | 2 | 17.89623 | 414 |
|  | **UHRF1** | 9.22E+13 | 43 | 0.5 | 46 | 1.93878 | 14.88943 | 344 |
|  | **WDHD1** | 9.22E+13 | 40 | 0.5 | 44.5 | 1.87755 | 10.32554 | 246 |
|  | **ZWINT** | 9.22E+13 | 41 | 0.5 | 45 | 1.89796 | 9.9593 | 246 |
